# Efficacy of prone positioning in awake ventilation for COVID-19: Umbrella review

**DOI:** 10.1097/MD.0000000000041477

**Published:** 2025-02-14

**Authors:** Dan-yang Guo, Qin Zhang, Li Wang, Zai-chun Pu, Ping Jia

**Affiliations:** a University of Electronic Science and Technology, Chengdu, China; b Sichuan Academy of Medical Sciences and Sichuan Provincial People’s Hospital, School of Medicine, University of Electronic Science and Technology of China, Chengdu, Sichuan, China; c Department of NICU, Sichuan Academy of Medical Sciences and Sichuan Provincial People’s Hospital, School of Medicine, University of Electronic Science and Technology of China, Chengdu, Sichuan, China.

**Keywords:** acute hypoxemic respiratory failure, acute respiratory distress syndrome, COVID-19, meta-analysis, prone position ventilation, protocol of awake prone position

## Abstract

**Background:**

Awake-prone positioning was widely used in COVID-19, combined with high-flow nasal oxygen therapy or noninvasive ventilation, effectively reducing intubation, and the effect on mortality is controversial. We aim to reevaluate the efficacy of awake-prone positioning in COVID-19 and summarize the protocol for awake-prone positioning.

**Methods:**

We gathered data on the treatment of COVID-19 using awake-prone positioning from Web of Science, Cochrane Library, Embase, PubMed, and CNKI. All the included studies were published between 2019 and 2023. Two researchers used the Assessment of Multiple Systematic Reviews tool to assess the methodological quality of the literature. The evidence was assessed using the Grading of Recommendations Assessment and Evaluation system.

**Results:**

Thirteen articles were included. The quality assessment using AMSTAR2 revealed that 3 articles were high quality, and 4 were moderate quality. The evidence quality assessment of 41 primary outcomes by the Grading of Recommendations Assessment, Development and Evaluation indicates that 9 indicators were of moderate quality, 21 were of low quality, and 6 were of very low quality.

**Conclusions:**

The review demonstrates high methodological quality, but the evidence quality of its outcome indicators is low. Awake-prone position has been shown to decrease intubation and improve oxygenation in COVID-19 patients. It is recommended to consult the latest quality assessment standards to develop more rigorous experimental protocols, improve research quality, and facilitate the translation of research findings.

## 1. Introduction

Patients with acute hypoxemic respiratory failure (AHRF) secondary to COVID-19 typically present with mild symptoms during the initial phases, which may progress to acute respiratory distress syndrome (ARDS) as the condition advances.^[1–3]^ ARDS^[4]^ is characterized by an inflammatory response, which can result in the development of pulmonary edema, hypoxemia, reduced lung compliance, and an increase in intrapulmonary shunting and dead space. According to reports, the mortality rate among patients diagnosed with AHRF and ARDS can reach 27% to 45%,^[5–7]^ with the majority requiring mechanical ventilation to improve respiratory function and maintain life. However, this may increase the risk of pulmonary infections^[6,8]^ and may also be associated with fatal outcomes.^[9]^ The guidelines^[10]^ suggest that prone ventilation has demonstrated a certain efficacy for patients with COVID-19 (moderate to severe ARDS); the prone position alters local pressure dynamics and enhances ventilation in poorly aerated lung regions, promoting lung recruitment and improving gas exchange.^[11]^ The timely implementation of prone positioning therapy has been shown to significantly decrease both the 28-day and 90-day mortality. During the COVID-19 pandemic, the prone position has been attempted in nonintubated patients with AHRF caused by COVID-19, the so-called awake-prone position (APP). Several randomized controlled trials (RCTs)^[12,13]^ have demonstrated the effectiveness in reducing the need for endotracheal intubation and improving the oxygenation index, but there is a diversity of opinions regarding its influence on mortality. We aim to conduct a systematic reevaluation and comprehensive assessment of the efficacy of APP ventilation for COVID-19.

## 2. Materials and methods

### 2.1. Study selection

The PICOS principle was used to establish the criteria for inclusion and exclusion. The inclusion criteria are as follows: conscious adult patients with COVID-19 (≥18 years old) who are not intubated; prone positioning in addition to standard care and/or other respiratory support methods; nonprone positioning in addition to standard care and/or other respiratory support methods; primary outcomes such as intubation rate, overall mortality rate, improvement in oxygenation index, etc; and RCTs, quasi-experimental studies, systematic reviews of observational studies, and meta-analyses. The exclusion criteria for this study include the following: literature that is not in Chinese or English; duplicate publications; conference abstracts, reports, or proposals with unextractable data or incomplete information; and low-quality literature with a total score of ≤ 4 on the Assessment of Multiple Systematic Reviews2 (AMSTAR2) methodological quality assessment tool.

### 2.2. Search strategy and data extraction

This systematic review adhered to Preferred Reporting Items for Systematic Reviews and Meta-Analyses (PRISMA) guidelines. The Cochrane Central Register of Controlled Trials, Web of Science, PubMed, and CNKI were systematically searched. The detailed search strategy is available in the supplementary materials (Table S1, Supplemental Digital Content, http://links.lww.com/MD/O376).

Two reviewers (GDY and JP) independently searched and screened study titles and abstracts, then reviewed the full texts of eligible studies for inclusion. Data were extracted on study characteristics (trial design, eligibility criteria, recruitment dates, number of centers, and countries); study population (age, sex, body mass index, severity, and hypoxemia status); type of care unit at enrollment; baseline oxygenation modality; descriptions of the trial intervention, control group, and co-interventions; and trial outcomes.

### 2.3. Study quality

Two authors evaluated each study included in the analysis to ascertain its quality. In cases where there were disagreements, these were resolved through discussion between the 2 authors, with the involvement of a third author if necessary. We evaluated the quality of observational cohort studies using the AMSTAR2 and the Grading of Recommendations Assessment, Development and Evaluation (GRADE) was utilized to assess the quality of evidence.

### 2.4. Date analysis

The study encompasses a wide range of intervention methods and assessment tools for outcome indicators, resulting in substantial heterogeneity across various studies. Furthermore, some studies failed to perform quantitative synthesis. Therefore, the present study uses a descriptive analysis methodology.

## 3. Results

### 3.1. Study selection

**Figure 1.**
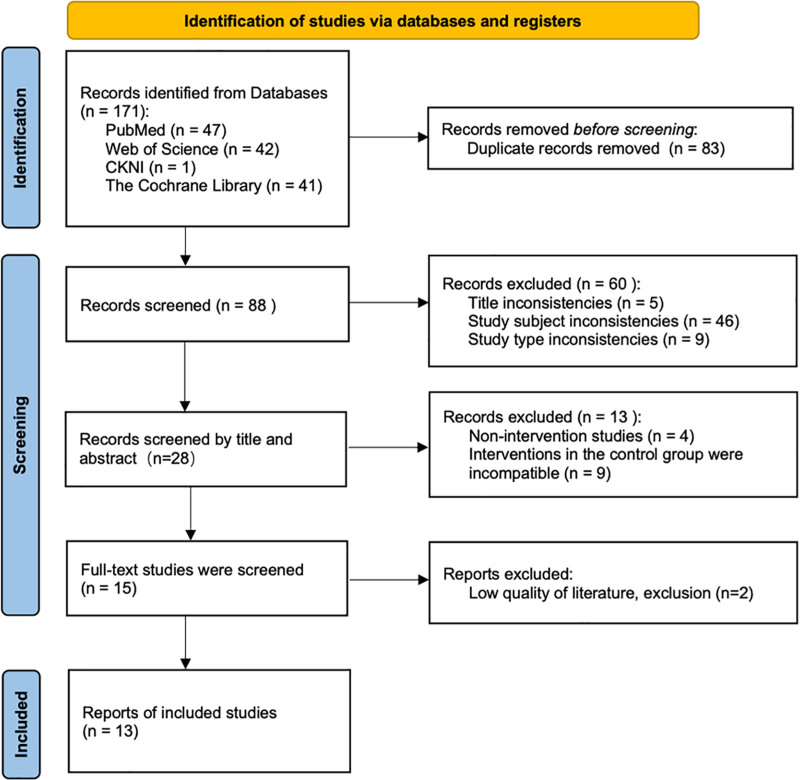
The PRISMA flow diagram of selected studies. PRISMA = Preferred Reporting Items for Systematic Reviews and Meta-Analyses.

A total of 13 English-language articles were collected, as shown in Figure 1.

### 3.2. Summary of study

The 13 studies included in this analysis^[14–26]^ were all English-language meta-analyses published between 2019 and 2022. The included studies consist of original research, including RCTs and nonrandomized controlled studies (non-RCTs), with sample sizes ranging from 243 to 5146 cases. The intervention measures in the treatment group primarily included APP and standard conventional treatment, along with some additional high-flow nasal cannula (HFNC) oxygen therapy and noninvasive ventilation (NIV) methods. The control group consisted of participants who were not placed in the prone position (other supine positions) and received standard conventional treatment. The methodological quality assessment tools used in the included studies varied. Seven studies^[15,18–20,22,24,25]^ used the Cochrane risk of bias assessment tool, 1 study^[26]^ used a mixed methods assessment tool, 5 studies^[15,17,19,21,25]^ used the Newcastle Ottawa Scale score, 1 study^[16]^ used the Qualsyst tool, and 1 study^[23]^ used the Jadad composite scale. The characteristics of the literature included are shown in Table 1.

**Table 1:** Characteristics of included studies (n = 13).

Authors	Country	Population	Study design	No. of participants	Protocol		Outcomes	Methodological quality assessment results
					Control	Intervention		
Ehrmann (2021)^[14]^	Canada, France, Ireland, Mexico, USA, Canada, France	COVID-19 nonendotracheal intubated adults (>18 yr) with respiratory failure	RCT	6 (1121)	Conventional standard recumbent therapy	Awake prone position	Endotracheal intubation rate and case fatality rate within 28 d	NA
Li (2022)^[15]^	Ireland, Mexico, USA, Spain, Sweden, Switzerland, India, Egypt, Qatar	COVID-19 nonendotracheal intubated adults (>18 yr) with respiratory failure	RCT	29 (4684)	Supine position	Awake prone position	The rate of tracheal intubation	The Cochrane Collaboration Risk of Bias tool/NOS
Pb (2021)^[16]^	New York, Italy, USA, Milan, France, Iran, Singapore, China	COVID-19 nonendotracheal intubated adults (>18 yr) with respiratory failure	NRSI	16 (316)	Conventional standard recumbent therapy	Awake prone position + NIV	The rate of tracheal intubation	The Qualsyst tool
Tan (2021)^[17]^	Denmark, Copenhagen, Singapore, China, US, Italy, Brazil, Mexico	Adult (≥18 yr old) awake patients with AHRF or ARDS	NRSI	16 (243)	NIV+Usual recumbent position	Awake prone position + NIV	The rate of tracheal intubation, and mortality	NOS
Schmi (2022)^[18]^	UK, Italy, India, Sweden, Canada, France, Ireland, Mexico, USA, Spain	COVID-19 causes respiratory failure in adults, necessitating HFNC, NIV or MV	RCT	5 (2182)	Supine or lateral decubitus position	Awake prone position (or prone position 135°)	All-cause mortality	The Cochrane Collaboration Risk of Bias tool
Fazzini (2022)^[19]^	Italy, China, USA, France, Spain, Brazil, Mexico, UK, Sweden	COVID-19 patients with AHRF require NIV	RCT, NRSI	14 (2352)	Usual recumbent position	Awake prone position + NIV	The change in oxygenation	The Cochrane Collaboration Risk of Bias tool/NOS
Chong (2022)^[20]^	Canada, France, USA, Mexico, Switzerland, Sweden, Spain	COVID-19 nonendotracheal intubated adults (>18 yr) with respiratory failure	RCT, NRSI	8 (1631)	Conventional standard recumbent therapy	Awake prone position	All-cause mortality and intubation rate	The Cochrane Collaboration Risk of Bias tool
Cardona (2021)^[21]^	USA, Italy, France, Iran, Singapore, China, Israel, Brazil	COVID-19 adult nonintubated patients in an awake state	NRSI	18 (364)	Conventional standard recumbent therapy + NIV	Awake prone position + NIV	Intubation rate	NOS
Weatherald (2022)^[22]^	Canada, USA, Arabia, France, Ireland, Mexico, Spain, Iran, Qatar, India, Switzerland	COVID-19 adults (≥18 yr old) in an awake state require HFNC and P/F < 300	RCT	17 (2931)	Usual or Conventional standard care	Awake prone position	Intubation rate	The Cochrane Collaboration Risk of Bias tool
Beran (2022)^[23]^	USA, Europe (Italy, France, Spain, and Sweden), Asia, South America	COVID-19 adult nonintubated patients in an awake state	RCT, NRSI	14 (3324)	Usual care	Awake prone position	The rate of tracheal intubation and mortality	The Jadad scale/NOS
Huang (2022)^[24]^	China	COVID-19 adults (>18 yr old) in ICU patients	RCT	10 (1686)	Supine position	Awake prone position	The rate of tracheal intubation	The Cochrane Collaboration Risk of Bias tool
Kang (2022)^[25]^	China	COVID-19 nonendotracheal intubated adults (>18 yr) with ARDS	RCT, NRSI	22 (5146)	Conventional standard recumbent therapy + NIV	Awake prone position + NIV	The rate of tracheal intubation and mortality	The Cochrane Collaboration Risk of Bias tool
Behesht (2021)^[26]^	France, Italy, Spain, USA, China, Germany, UK, Brazil, Turkey, Canada	COVID-19 adult nonintubated patients require NIV	NRSI	54 (1272)	Usual care	Awake prone position (≥30–60 min)	The respiratory parameters and respiratory status	MMAT

ICU = intensive care unit, MMAT = mixed methods assessment tool, NIV = noninvasive ventilation, NOS = Newcastle Ottawa Scale, NRSI = non-randomised studies of the effects of interventions, RCT = randomized controlled trial.

## 4. Study quality

### 4.1. Methodological quality assessment results included in the literature

The results of the AMSTAR2 quality assessment in this article show that there are 4 medium-quality studies,^[15,19,21,25]^ 7 low-quality studies,^[16–20,23,26]^ and 3 high-quality studies.^[14,22,24]^ The included studies demonstrate a high level of methodological quality. The evidence quality of the outcome indicators in the included studies was evaluated using the GRADE system. The evaluation results of 41 primary outcome indicators from 13 systematic reviews using the GRADE system showed that 9 pieces of evidence were of moderate quality, 21 were of low quality, and 6 were of very low quality. Item 2 has 4 studies^[16,17,21,23]^ marked as “no”; the rest are marked as “yes.” Most of the included articles provide complete research plans and are registered on relevant platforms. There is considerable controversy surrounding item 3, as the included studies have not provided a strong explanation for the selection of the study types to be included. Item 7 has only 1 study^[24]^ that is marked as “yes,” providing a complete list of excluded studies and the relevant reasons. Item 10 has only 1 study^[14]^ marked as “yes” due to its unique article type, as other studies did not specify the funding sources for the original research. The results of the methodological quality assessment for the included studies are shown in Table 2. In the Pb et al,^[16]^ item 3 was marked as “no” as the study protocol was not preregistered at PRISMAR. The list of excluded literature and the reasons for exclusion were required by item 7 but not listed, and the process and results of the risk of bias detection were not listed in the process of analyzing the articles, so items 12 and 13 were both no, and the quality of this meta-analysis was marked as very low.

**Table 2:** Quality assessment of studies using AMSTAR (n = 13).

	①	②	③	④	⑤	⑥	⑦	⑧	⑨	⑩	⑪	⑫	⑬	⑭	⑮	⑯	Credibility
Ehrmann^[14]^	Y	Y	Y	PY	Y	Y	Y	Y	Y	Y	Y	Y	Y	Y	Y	Y	H
Li^[15]^	Y	Y	PY	PY	Y	Y	PY	Y	Y	N	Y	N	Y	Y	Y	Y	M
Pb^[16]^	Y	N	PY	PY	Y	Y	PY	Y	Y	N	Y	N	N	PY	PY	Y	VL
Tan^[17]^	Y	N	PY	PY	Y	Y	PY	Y	Y	N	Y	N	Y	Y	Y	Y	L
Schmid^[18]^	Y	Y	PY	PY	Y	Y	PY	Y	Y	N	Y	Y	Y	N	PY	Y	L
Fazzini^[19]^	Y	Y	PY	PY	Y	Y	PY	Y	Y	N	Y	N	Y	Y	PY	Y	M
Chong^[20]^	Y	Y	PY	PY	Y	Y	PY	Y	Y	N	Y	N	Y	N	N	Y	VL
Cardona^[21]^	Y	N	PY	Y	Y	Y	PY	Y	Y	N	Y	Y	Y	Y	PY	Y	M
Weatherald^[22]^	Y	Y	PY	PY	Y	Y	PY	Y	Y	N	Y	Y	Y	Y	Y	Y	H
Beran^[23]^	Y	N	N	PY	Y	Y	PY	Y	Y	N	Y	N	Y	Y	Y	Y	L
Huang^[24]^	Y	Y	PY	PY	Y	Y	Y	Y	Y	N	Y	Y	Y	Y	Y	Y	H
Kang^[25]^	Y	Y	PY	PY	Y	Y	PY	Y	Y	N	Y	Y	Y	Y	Y	Y	M
Behesht^[26]^	Y	Y	PY	Y	Y	Y	PY	Y	PY	N	Y	N	N	Y	Y	Y	L

H = high, L = low, M = medium, N = no, NP = no performed, PY = partial yes, VL = very low, Y = yes.

### 4.2. Quality assessment results of evidence included in the literature

A total of 13 systematic evaluations/meta-analyses included 7 outcome indicators and 41 pieces of evidence. Among them, 9 were graded as moderate according to the GRADE system, 21 were graded as low, and 6 were graded as very low. The quality assessment results of the included studies are available in Table 3.

**Table 3:** GRADE quality of evidence results.

Outcomes	Limitations	Inconsistency	Indirectness	Imprecision	Publication bias	Quality
Ehrman (2021)^[14]^						
Treatment failure at day 28 (intubation or death)	0	0	0	0	0	H
Intubation rate at day 28	−1*	0	0	−1§	0	L
Mortality at day 28 (all patients)	0	0	0	−1§	−1∥	L
Mortality at day 28 (invasively mechanically ventilated patients)	0	0	0	−1§	0	M
Li (2022)^[15]^						
Intubation rate	0	0	0	0	0	H
All-cause mortality	0	−1†	0	0	0	M
Escalation of oxygen modality	−1*	−1†	0	0	0	L
ICU length of stay	−1*	0	0	0	0	M
Hospital length of stay	0	−1†	0	0	0	M
Pb (2021)^[16]^						
Intubation rate	−1*	0	0	−1§	−1∥	VL
Improvement of oxygenation	−1*	0	0	−1§	0	L
Tan (2021)^[17]^						
Intubation rate and mortality	0	0	0	−1§	−1∥	L
Improvement of oxygenation	−1*	−1†	0	−1§	0	VL
Intolerance rate	0	−1†	0	−1∥	0	L
Schmid (2022)^[18]^						
All-cause mortality at day 28, day 60	0	0	0	−1§	−1∥	L
Duration of hospitalization	0	−1†	0	0	−1∥	L
Serious adverse events during the study period	−1*	0	0	−1§	0	L
Hospital length of stay	−1*	0	0	0	−1∥	L
Fazzini (2022)^[19]^						
Improvement of oxygenation	−1*	0	0	−1§	0	L
Intubation rate	0	−1‡	0	−1§	−1∥	VL
Mortality	0	−1†	0	−1§	0	L
Chong (2022)^[20]^						
All-cause mortality and intubation rate	0	0	0	0	0	H
Improvement of oxygenation	−1*	−1‡	0	−1§	0	VL
Hospital length of stay	−1*	−1‡	0	0	0	L
Cardona (2021)^[21]^						
Intubation rate	−1*	−1†	0	−1§	0	VL
the rate of intubation within 24 h of and mortality rate	−1*	0	0	−1§	0	L
Weatherald (2022)^[22]^						
Intubation rate	0	0	0	0	0	H
Mortality	−1*	0	0	0	0	M
Hospital length of stay	−1*	0	0	−1§	0	L
Improvement of oxygenation	0	−1†	0	−1§	0	L
Beran (2022)^[23]^						
Intubation rate	0	0	0	0	0	H
Mortality	0	−1†	0	0	0	M
Hospital length of stay	−1*	−1†	0	0	0	L
Huang (2022)^[24]^						
Intubation rate in ICU	−1*	0	0	0	0	M
All-cause mortality at the longest follow-up available	0	0	0	−1§	0	M
ICU length of stay	−1*	−1‡	0	0	0	L
Kang (2022)^[25]^						
Intubation rate	−1*	−1‡	0	0	0	L
Mortality	0	−1‡	0	0	0	M
Behesht (2021)^[26]^						
Improvement of oxygenation	−1*	−1‡	0	0	0	L
Mortality	0	−1‡	0	−1§	0	L
Intubation rate	−1*	−1†	0	−1§	0	VL

Risk of bias. GRADE = Grading of Recommendations Assessment, Development and Evaluation, L = low, M = moderate, VL, very low.

* Significant risk of bias in the inclusion of studies in terms of randomization, blinding, allocation concealment, integrity of outcome data, or selective reporting.

High risk of bias in the inclusion of studies in terms of randomization, blinding, allocation concealment, integrity of outcome data, or selective reporting.

† 50% ≤ I² < 75%.

‡ 75% ≤ I² ≤ 100% after the combination of the included data.

§ Sample size was small (continuity variable < 400, dichotomies < 300) or 95% CI crossed the invalid line.

∥ The funnel plot was asymmetric or consistently positive results are present.

## 5. Outcomes

### 5.1. Intubation rate and mortality

Twelve studies^[14–25]^ have investigated the efficacy of APP with the primary outcomes of endotracheal intubation rates and mortality rates. Most studies provide evidence in favor of a decrease in endotracheal intubation rates following APP interventions, although there is controversy regarding the impact on mortality outcomes. Kang et al^[25]^ initially discovered a significant correlation between APP and decreased intubation and mortality rates in cases of AHRF or ARDS related to COVID-19. Importantly, without any concurrent rise in adverse events, upon conducting subgroup analysis, no statistically significant difference in mortality was observed, which aligns with the conclusions drawn by Weatherald et al.^[20,22]^ According to Behesht et al,^[26]^ the lower mortality and intubation rates may be attributed to the improvement in patients’ oxygenation. Early prone positioning, along with HFNC or NIV, significantly reduces intubation rate in patients with moderate to severe ARDS (even in non-COVID-19 patients). The study incorporating the use of a prone position combined with HFNC as the intervention group also yielded comparable findings.^[27]^ However, Li et al^[15]^ have acknowledged a significant decrease in the rate of tracheal intubation, but through subgroup analysis, they have refuted the association between the risk of intubation and the site of enrollment, as well as the distinction between various respiratory support methods (intensive care unit [ICU] vs non-ICU) (advanced respiratory support vs conventional respiratory support).

Notably, a decrease in mortality was observed by Fazzini et al,^[19]^ with no change in the intubation. After excluding studies with high heterogeneity, Beran et al^[23]^ similarly concluded that APP may reduce the in-hospital mortality rate of COVID-19 patients but does not have a significant impact on intubation rate or length of hospital stay. Furthermore, Huang et al^[24]^ have considered that the decrease in intubation rates associated with APP appears to be related to its duration. They propose that patients with more severe conditions in ICU COVID-19 (ie, requiring HFNC or NIV, average SPO_2_/FiO_2_ < 150 mm Hg, or higher risk of mortality), obesity, or those aged 70 years and above are more inclined to derive benefits from APP. Several studies have indicated that the transient changes in oxygenation observed following APP do not significantly impact patient outcomes. Many original studies have reported challenges in attaining the desired duration of prone positioning. Ehrmann et al^[14]^found no significant variations in intubation or mortality rates following APP intervention for a follow-up period of 28 days or more. This conclusion aligns with Tan et al^[17]^ reached a similar conclusion.

### 5.2. Oxygenation index

Six studies^[16,17,19,20,22,26]^ evaluated the efficacy of APP in terms of the PaO2/FiO2 ratio, SPO2/FiO2, respiratory rate, or respiratory status. The results showed that APP can enhance the oxygenation index of patients. Tan et al^[17]^ found that the use of APP can significantly improve oxygenation and reduce respiratory rate in awake patients with AHRF or ARDS. Both COVID-19 and non-COVID-19 patients can benefit from it. However, Behesht et al^[26]^ contradicted the improvement of respiratory rate as reported in their study. From a pathophysiological perspective, this study explains how prone positioning improves the oxygenation index.^[19]^ In cases of viral pneumonia (such as SARS-CoV-2 or influenza) or ARDS,^[28,29]^ the enhancement of oxygenation is strongly associated with a more even distribution of ventilation in the dorsal lung regions. This leads to effective lung recruitment and a notable increase in the ventilation-perfusion ratio (V/Q). The study conducted by Chong et al^[20]^ did not find any statistically significant differences in oxygen and index changes among patients following APP treatment. In a study comparing the APP group to the conventional treatment group, no statistically significant differences were found in terms of oxygen delivery as the outcome measure.^[22]^

### 5.3. Hospital length of stay

Seven studies^[14,15,18,20,22–24]^ collectively examined the influence of the APP on the length of hospital stay as a secondary outcome. In the RCTs,^[14]^ there was no statistically significant difference in the average length of hospital stay between the intervention group and the control group.^[18,20,23,26]^ The use of the APP did not have a significant impact on the length of hospital stays for patients. In the non-ICU patient group of the APP team, a small but statistically significant difference in length of hospital stay was found. Similar conclusions were drawn in the observational study.

### 5.4. Escalation of oxygen modality

Three studies^[15,17,22]^ analyzed the improvement of oxygen support methods and their impact on secondary outcomes. The change in respiratory support methods had no impact on patients. In the 7 RCTs included by Li et al,^[15]^ no need to change the respiratory support method was found in the patients. Weatherald et al^[22]^ reached a similar conclusion in a study involving 1611 patients.

### 5.5. Adverse events

In the 13 studies analyzed, common adverse events included tube dislodgement and pressure injuries. Awake patients had a higher incidence of intolerance. The prevalence of adverse events is generally low. Two studies^[19,25]^ have indicated that patients experienced serious adverse events, such as cardiac arrest, whereas other studies have not documented any such events. The assessment and comparison of the level of comfort and breathlessness before and during the awakening of the Pb et al^[16]^ were conducted using the visual analog scale. The score for breathlessness showed improvement, decreasing from 3 to 2, whereas the score for discomfort increased from 0 to 4. The study^[24]^ examines adverse events as secondary outcomes. The occurrence of adverse events resulting from the APP is minimal, and both independent analyses of RCTs and observational studies have shown no significant disparity in the incidence of adverse events between the group receiving the APP and the control group.

## 6. Discussion

### 6.1. Principal findings and possible explanations

APP used as an effective intervention to improve patient oxygenation and respiration has demonstrated a substantial decrease in mortality rate and intubation rate among individuals with severe ARDS patients.^[10]^ The application of prone positioning in patients with COVID-19 has once again garnered recognition from a multitude of healthcare professionals. In nonintubated patients with AHRF caused by COVID-19, the early combined use of HFNC or NIV respiratory support can effectively reduce the need for endotracheal intubation. The mechanism of severe pneumonia can be used to explain this phenomenon.^[30,31]^ When patients assume a prone position, the influence of gravity causes changes in local pressure and gradient, resulting in the reopening of poorly ventilated or nonventilated areas of the lungs, thus promoting lung recruitment. When patients with autonomous respiration are placed in the prone position, the changes in pleural pressure and distribution of the pleural space across the entire lung area promote uniform ventilation and lung expansion, demonstrating the effectiveness of short-term intervention in this conclusion.

We conducted a systematic review and meta-analysis to evaluate the efficacy of APP in the treatment of AHRF. The results suggest that the administration of APP has benefits in terms of reducing the requirement for endotracheal intubation, mortality rates, and enhancing the oxygenation index among these individuals. The study also found that there were no statistically significant variations in the duration of hospital stay and the approach to respiratory support between the APP and traditional standard treatments. On one hand, the observed limitations in this study can be attributed to the inadequate sample size of patients and the absence of blinding among healthcare personnel during the study inclusion process, which may have produced a certain bias in the assessment of patient status and the determination of intubation requirements. Patients will also receive additional medical advice from the doctor based on the progression of their illness. The lack of standardized criteria for the initiation of invasive mechanical ventilation may impact clinical decision-making, as the threshold for initiating invasive mechanical ventilation in the standard treatment group is comparatively low. Some studies do not approve that benefits for patients may derive from delayed intubation decisions,^[32,33]^ thereby giving rise to concerns regarding the safety and feasibility of postponing intubation.^[34]^ Ibarra-Estrada et al^[35]^ aimed to identify factors for the success of individuals benefiting from the prone position, identify sensitive populations, and improve the feasibility and effectiveness of APP intervention measures. The establishment of standardized guidelines for tracheal intubation and the standardization of the assessment process for tracheal intubation necessitate additional efforts. On the contrary, numerous studies have failed to establish a precise definition for the duration of prone positioning, leading to significant regional discrepancies. The adherence to prone positioning among awake patients who have not been administered sedative-related medications is suboptimal, and these patients report a lower subjective tolerance for the discomfort associated with prone positioning.^[36,37]^ Additionally, most studies concentrate on APP, where patients independently decide whether to initiate the use of the APP. Patients often amplify their discomfort based on subjective thoughts. In these studies, the duration of supine positioning frequently does not meet established standards, and the premature termination of APP requires attention. The APP implementation is overseen by medical professionals who offer support and encouragement to patients. This could be an effective way to improve the effectiveness of the app. To enhance the research plan, it is possible to explore the perspective of setting prone position time, objectively evaluating intolerance, and involving qualified personnel to assess the patient’s current condition before proceeding with additional treatments.

In the current investigation, the researchers utilized AMSTAR2 as a methodology to evaluate the included research. The results indicated that the methodological quality of 4 systematic reviews/meta-analyses was deemed to be of low quality, as evidenced by the presence of significant information missing in items 3, 7, and 10. In terms of recommendations for future research: the lack of blinding in RCTs can be achieved by enhancing the training of assessors and ensuring the rigor and standardization of RCT protocol design. Additionally, it is recommended that RCTs be registered and made accessible to the public through established experimental platforms. In the screening phase of the included studies, it is crucial to reduce the subjective influence on the process of article selection by adding the exclusion criteria and checklist. The inclusion of various research types in a study can have an impact on the outcomes of research objectives. Therefore, it is of utmost importance to meticulously deliberate the selection of research methodologies to prevent the inadvertent preference of low-quality RCTs over high-quality observational studies.

From 13 studies, a total of 41 outcomes were collected, consisting of 5 high-level evidence and 9 moderate-level evidence. Most outcomes with higher levels of evidence were obtained from studies characterized by high original research quality or perfect statistical analysis methods and a relatively low presence of publication bias. The GRADE evidence grading system was used to score. Most studies exhibit deficiencies in blinding, inconsistent outcome measures, insufficient sample sizes, and uncertainty regarding the reliability of confidence intervals when graded for outcome measures. The research can utilize evaluation criteria to enhance the quality of its content. High-quality original studies, especially RCTs, play a crucial role in generating reliable evidence for meta-analyses and contribute to the advancement of evidence-based guidelines that can be effectively translated into clinical practice, thus offering valuable assistance to healthcare professionals in their clinical work.

### 6.2. Limitations of the study

The research lacks methodological quality, as it does not perform sensitivity analysis on studies with high bias. Some of the primary literature included in the study lacks sufficient case numbers to support the results and lacks high credibility in the findings. The studies generally do not mention consulting professionals in the relevant field, which may be one of the reasons affecting the quality of the articles.

## Acknowledgments

During the preparation of this study, the authors used WORDIVCEAI to Polish the article. After using this tool/service, the author(s) reviewed and edited the content as needed and took full responsibility for the content of the publication.

## Author contributions

**Data curation:** Dan-yang Guo, Qin Zhang.

**Methodology:** Dan-yang Guo, Qin Zhang.

**Validation:** Dan-yang Guo.

**Writing—original draft:** Dan-yang Guo, Li Wang.

**Writing—review & editing:** Dan-yang Guo.

**Visualization:** Li Wang.

**Software:** Zai-chun Pu.

**Supervision:** Zai-chun Pu, Ping Jia.

**Conceptualization:** Ping Jia.

**Project administration:** Ping Jia.

## Supplementary Material


