# Supplementary material for: Efficacy of prone positioning in awake ventilation for COVID-19: Umbrella review
Source: Medicine (Baltimore). 2025 Feb 14;104(7):e41477. doi: 10.1097/MD.0000000000041477 (PMC11835137; doi:10.1097/MD.0000000000041477)
Supplement: Supplementary file 1 [file medi-104-e41477-s001.docx]

| **PubMed** | **Search number** | **Query** | **Results** |  |
| --- | --- | --- | --- | --- |
|  | **4** | (((((((((((((((COVID-19[MeSH Terms]) or (covid*[Title/Abstract])) OR (SARS-CoV*[Title/Abstract])) OR (coronavirinae*[Title/Abstract])) OR (SARS-CoV*[Title/Abstract])) OR (SARSCoV*[Title/Abstract])) OR (SARS-corona*[Title/Abstract])) OR (nCoV*[Title/Abstract])) OR (n-CoV*[Title/Abstract])) OR (novel CoV*[Title/Abstract])) OR (2019-nCoV*[Title/Abstract])) OR (2019nCoV*[Title/Abstract])) OR ("severe acute respiratory syndrome coronavirus 2"[Title/Abstract])) OR ("severe acute respiratory syndrome CoV 2"[Title/Abstract])) AND ((((prone position[MeSH Terms]) OR (proned[Title/Abstract])) OR (proning[Title/Abstract])) OR (self-pron*[Title/Abstract]))) AND ((((("meta-analysis"[Title/Abstract] OR [Title/Abstract]) OR ("Meta analysis as Topic"[Title/Abstract])) OR ("systematic review"[Title/Abstract])) OR ("meta analysis"[Title/Abstract])) OR ("meta analyses"[Title/Abstract])) | 47 |  |
|  | **3** | (((("meta-analysis"[Title/Abstract] OR [Title/Abstract]) OR ("Meta analysis as Topic"[Title/Abstract])) OR ("systematic review"[Title/Abstract])) OR ("meta analysis"[Title/Abstract])) OR ("meta analyses"[Title/Abstract]) | 482,971 |  |
|  | **2** | (((prone position[MeSH Terms]) OR (proned[Title/Abstract])) OR (proning[Title/Abstract])) OR (self-pron*[Title/Abstract]) | 5,542 |  |
|  | **1** | (((((((((((((COVID-19[MeSH Terms]) or (covid*[Title/Abstract])) OR (SARS-CoV*[Title/Abstract])) OR (coronavirinae*[Title/Abstract])) OR (SARS-CoV*[Title/Abstract])) OR (SARSCoV*[Title/Abstract])) OR (SARS-corona*[Title/Abstract])) OR (nCoV*[Title/Abstract])) OR (n-CoV*[Title/Abstract])) OR (novel CoV*[Title/Abstract])) OR (2019-nCoV*[Title/Abstract])) OR (2019nCoV*[Title/Abstract])) OR ("severe acute respiratory syndrome coronavirus 2"[Title/Abstract])) OR ("severe acute respiratory syndrome CoV 2"[Title/Abstract]) | 452,635 |  |
| **Web of science** | **Search number** | **Query** | **Results** |  |
|  | 1 | (((((((((((((TS=(covid*)) OR TS=(coronavirus*))) OR TS=(coronavirinae*)) OR TS=(SARS-CoV*)) OR TS=(SARSCoV*)) OR TS=(SARS-corona*)) OR TS=(nCoV*)) OR TS=( n-CoV*)) OR TS=(novel CoV* )) OR TS=(2019-nCoV*)) OR TS=(2019nCoV*)) OR TS=("severe acute respiratory syndrome coronavirus 2")) OR TS=("severe acute respiratory syndrome CoV 2") | 498569 |  |
|  | 2 | ((((TS=("meta-analysis")) OR TS=( "Meta analysis as Topic")) OR TS=("systematic review")) OR TS=( "meta analysis" )) OR TS=("meta analyses") | 443167 |  |
|  | 3 | (((TS=(proned )) OR TS=( proning )) OR TS=(self-pron*)) OR TS=(prone position) | 10216 |  |
|  | 4 | #3 AND #2 AND #1 | 42 |  |
| **The Cochrane library** | **Search number** | **Query** | **Results** |  |
|  | 1 | MeSH descriptor: [COVID-19] explode all trees | 8488 |  |
|  | 2 | (covid*):ti,ab,kw OR (coronavirus*):ti,ab,kw OR (coronavirinae*):ti,ab,kw OR (SARS-CoV*):ti,ab,kw OR (SARSCoV*):ti,ab,kw | 23807 |  |
|  | 3 | (SARS-corona*):ti,ab,kw OR (novel CoV*):ti,ab,kw OR (nCoV*):ti,ab,kw OR (n-CoV*):ti,ab,kw OR (severe acute respiratory syndrome coronavirus 2):ti,ab,kw | 7110 |  |
|  | 4 | (severe acute respiratory syndrome CoV 2):ti,ab,kw | 303 |  |
|  | 5 | #2 or #3 or #4 | 26196 |  |
|  | 6 | MeSH descriptor: [SARS-CoV-2] explode all trees | 3647 |  |
|  | 7 | MeSH descriptor: [Coronavirus] explode all trees | 3687 |  |
|  | 8 | #1 or #5 or #6 or #7 | 26197 |  |
|  | 9 | MeSH descriptor: [Prone Position] explode all trees | 499 |  |
|  | 10 | (pron*):ti,ab,kw OR (position*):ti,ab,kw OR (self-pron*):ti,ab,kw | 65758 |  |
|  | 11 | #9 or #10 | 65758 |  |
|  | 12 | (meta-analysis):ti,ab,kw OR (Meta analysis as Topic):ti,ab,kw OR (systematic review):ti,ab,kw OR (meta analysis):ti,ab,kw OR (meta analyses):ti,ab,kw | 30410 |  |
|  | 13 | #8 and #11 and #12 | 41 |  |

**Supplementary Table 1 Search strategies**
